# Usage of the Tablet-Based “Keep On Keep Up” Digital Program and Resulting Changes in Physical Capacity and Real-World Walking in Community-Dwelling Older Adults: Process Evaluation

**DOI:** 10.2196/80372

**Published:** 2026-05-13

**Authors:** Melissa J Böttinger, Marios Stefanakis, Tobias Eckert, Carl-Philipp Jansen, Elena Litz, Anastasia Bredenbrock, Sabato Mellone, Leon Radeck, Anna-Lena Schubert, Tim Fleiner, Simon Steib, Emma Stanmore, Chris Todd, Jürgen M Bauer, Clemens Becker, Katharina Gordt-Oesterwind

**Affiliations:** 1 Geriatric Center, Medical Faculty Heidelberg, Heidelberg University, Bergheimer Str. 20, Heidelberg, 69115, Germany, 49 17683011750; 2 Network Aging Research, Heidelberg University, Heidelberg, Germany; 3 Institute for Geriatric Research, Ulm University Medical Center, Ulm, Germany; 4 Department of Electrical, Electronic and Information Engineering "Guglielmo Marconi", Università di Bologna, Bologna, Italy; 5 Institute for Computer Science, Heidelberg University, Heidelberg, Germany; 6 Department of Psychology, Johannes Gutenberg University Mainz, Mainz, Germany; 7 Institute of Medical Engineering and Mechatronics, Ulm University of Applied Sciences, Ulm, Germany; 8 Institute of Sports and Sports Sciences, Heidelberg University, Heidelberg, Germany; 9 School of Health Sciences, Faculty of Biology, Medicine and Health, The University of Manchester, Manchester, United Kingdom; 10 National Institute for Health and Care Research, Applied Research Collaboration – Greater Manchester (NIHR ARC-GM), Manchester, United Kingdom; 11 Manchester Academic Health Science Centre, Manchester, United Kingdom; 12 Manchester University NHS Foundation Trust, Manchester, United Kingdom

**Keywords:** digital intervention, mobile apps, exercise therapy, physical capacity, real-world walking, treatment adherence and compliance, aged

## Abstract

**Background:**

“Keep On Keep Up” (KOKU) is a tablet-based digital program based on the well-validated Otago and Fitness and Mobility Exercise programs for older adults to decrease the risk of falling.

**Objective:**

This substudy involved a process evaluation in order to analyze the usage patterns of the KOKU digital program, specifically training frequency, volume, and intensity among older adults over a 3-month self-managed training period. Pre-post changes in physical capacity and real-world walking were examined.

**Methods:**

This study is a nested cohort study within the three-armed randomized controlled SMART-AGE trial conducted in Germany (German Clinical Trials Register ID: DRKS00034316). Participants aged 67 years or older with basic digital literacy were included. KOKU provided guided but unsupervised progressive strength and balance training for 3 months. The data on training adherence, engagement, and progression were collected. Instrumented assessments included the Timed Up and Go Test, the 30-Second Chair Rise Test, and real-world walking monitoring using wearable sensors.

**Results:**

A total of 113 participants (n=63, 56% female; mean age 74.02, SD 5.36 y) were included in the analysis. During the 3-month period, participants used KOKU for 24 (SD 15) days, that is, 2 to 3 times per week. Over the entire study period, no falls or other adverse events were reported due to KOKU usage. The number of exercises performed per participant ranged from 2 to 213, with a median value of 70. The instrumented Timed Up and Go Test results revealed a prolonged total duration (*d*=0.26; *P*=.009). In the instrumented 30-Second Chair Rise Test, improvements were observed in the number of completed repetitions (*d*=0.21; *P*=.04) and frequency of repetitions (*d*=0.23; *P*=.03). This was mainly due to a reduction in inactive time (*d*=−0.60; *P*<.001). Real-world walking parameters remained unchanged, except for a slower walking speed during walking bouts of less than 30 seconds (*d*=0.49; *P*<.001). All changes did not meet the criteria for minimally important differences.

**Conclusions:**

KOKU is a novel digital intervention for older adults, promoting balance and strength exercises. Physical capacity improvements were small. However, the use of instrumented assessments provided further insights into participants’ capacity and mobility that would not have been identifiable with conventional assessments. Future improvements to the program should focus on incorporating more challenging exercises for individuals with varying levels of physical capacity.

## Introduction

Physical activity and exercise are vital cornerstones for older adults’ mobility, independence, and quality of life [1]. With increasing age, functional limitations and decline become more common due to the loss of muscle mass, reduced bone density, slower reaction times, and impaired balance control, highlighting the importance of effective exercise and training programs that support the maintenance of mobility and independence [2,3].

Traditional exercise programs, such as the Otago Exercise Programme [4], have been shown to reduce fall risk and improve strength and balance [5]. However, growing challenges posed by health care workforce shortages and demographic shifts make timely access to primary care and subsequent referrals for further diagnostics or therapy increasingly difficult [6]. Further factors hindering people from attending training are low availability of trainers or therapists, limited local transport in rural or remote areas, and extreme weather conditions, such as heat or snow [7,8].

These challenges highlight the need for training programs that older people can access independently, safely, and autonomously from home. Digital technologies offer promising opportunities to bridge impending gaps in health care and provide convenient, scalable, and accessible options for older adults to engage in physical activity and training, especially balance and strength exercises to improve mobility, prevent falls, and maintain independence [9,10]. Different tools, such as mobile apps, can provide tailored training interventions, making it easier for older adults to incorporate regular training into their daily lives [11].

The “Keep On Keep Up” (KOKU) tablet-based digital program is designed specifically for older adults at risk of falling [12]. It is based on the Otago [4] and the Fitness and Mobility Exercise programs [13] and addresses physical capacity to prevent falls. KOKU is an evidence-based training program that is recommended for use for a period of about 3 months or longer and incorporates safety features for independent training at home [14]. It includes balance and strength exercises that progress in difficulty from sitting to standing and walking within the training period. Feasibility has been tested with participants in different countries, and KOKU has received high usability and acceptability scores in previous studies [15]. However, evidence on how digital interventions, and especially KOKU, are used independently by older adults over an extended period, and what potential physical changes may result from its use in a real-world context remains limited [16]. Despite the potential of digital interventions for independent training, challenges such as limited digital literacy, usability concerns, and motivational factors may affect adherence and outcomes in older adults [17].

To capture possible changes following KOKU usage, assessments giving detailed insights into physical capacity and mobility are essential. Instrumented assessments offer significant advantages over traditional methods by capturing detailed movement data, such as the different segments of the Timed Up and Go Test [18], and informing training regimens and progress monitoring. For example, sensor-based assessments can provide additional insights into movement phases during functional mobility tests and may support a more precise evaluation of functional mobility.

For the future, the combination of instrumented assessments with digital interventions could represent a powerful approach to promoting physical activity and health among older adults. This synergy could enhance the personalization and effectiveness of training programs. Such a combination may enhance the monitoring of training progress and support the development of more personalized and adaptive training programs.

The aim of this process evaluation was to examine how older adults used the KOKU digital exercise program during a 3-month self-managed training period in terms of training frequency, volume, intensity, as well as safety, and to explore potential changes in physical capacity and real-world walking.

## Methods

### Study Design

This study is embedded as a nested cohort within the 3-armed randomized controlled trial SMART-AGE (P2019-01-003) conducted at Heidelberg University, Germany [19]. It is registered with the German Clinical Trials Register (DRKS00034316).

The process evaluation and its findings were reported following the CONPHES (Consensus-Based Process Evaluation Reporting Guideline for Public Health Intervention Studies) guideline, developed for public health interventions conducted alongside effectiveness trials [20].

### Participant Recruitment

Inclusion criteria for participation in the SMART-AGE study were age 67 years or older, living in the community, having at least basic knowledge of using PCs or tablets, and being German-speaking. Exclusion criteria were severe cognitive impairment, severe medical conditions (eg, Parkinson disease with use of a walker or wheelchair), and severe visual or hearing impairment.

### Ethical Considerations

The SMART-AGE study was approved by Heidelberg University Medical Faculty’s Ethical Committee (S-672/2022). All participants gave written, informed consent prior to participation. All data were collected and stored in pseudonymized form. Participants were allowed to keep the tablet used in the study as a form of compensation for their time and effort. All KOKU users were covered by a trial insurance during the study period.

### KOKU App

The tablet-based digital program “KOKU Health–Keep On Keep Up” is a strength and balance training program designed for older adults to prevent physical decline, frailty, and reduce the risk of falls [14]. After an initial introduction by a physiotherapist or qualified exercise specialist, users are able to undertake a structured exercise program independently at home without supervision. For this study, the KOKU program was translated into German by 2 native speakers, ensuring that its design, structure, and functionality were fully preserved. A professional voice-over was created for the explanatory videos to maintain the quality and accessibility of the content.

Before commencing the training program, participants completed an initial assessment within KOKU: the Short Falls Efficacy Scale-International (FES-I) [21], EQ-5D [22], and questions about their history of falls and general well-being. The FES-I appears automatically again after having reached the end of level 3 and level 6 to evaluate changes in perceived concern about falling. Additionally, participants are asked weekly to report any slips, trips, or falls, including their causes and consequences. The KOKU training program comprises 7 levels, each lasting a minimum of 2 weeks. Each week includes 3 training days, with 3 exercises per day. Participants were advised to perform 12 to 15 repetitions of each exercise, or as often as possible. Every exercise is accompanied by written instructions and video demonstrations with voice-overs, providing step-by-step guidance to ensure proper and safe execution. The initial level introduces simple exercises, such as heel raises while seated and sit-to-stand movements. After each exercise, users input the number of repetitions completed. Upon finishing a session, participants provide feedback on their physical exertion. Based on these data, the training progression is automatically adjusted and advances to higher levels with more challenging exercises, such as the tandem stance or walking backward. In addition to physical exercises, KOKU incorporates 4 health literacy games that address potential tripping hazards and promote healthy nutrition. Participants were encouraged to use KOKU 3 times per week over a 3-month period. KOKU sends automatic reminder messages after 1 week of inactivity to promote adherence.

Home visits for the introduction of KOKU and assessments were conducted by trained assessors with professional backgrounds in physiotherapy or sports science, following a standardized protocol to ensure consistency and reliability.

### Data Collection and Processing

#### Participant Data Collection

To describe the sample, baseline data were extracted from the SMART-AGE trial for the following sociodemographic and clinical characteristics: age, sex, BMI, living situation, employment status, Groll Functional Comorbidity Index [23], Short-FES-I [21], 6-item Cognitive Impairment Test [24], Trail Making Test [25], WHO Quality of Life Scale [26], and Mobile Device Proficiency Questionnaire [27]. The recording of past falls in the last 12 months was part of the initiation of KOKU.

#### Digital Monitoring Data of KOKU

Usage data, such as app openings, exercise sessions, gameplay, and questionnaire responses, were transmitted to a server. Upon transmission, the server receives and stores the data in a database for further analysis.

KOKU was designed to run in both online and offline environments. When an internet connection was available, data were transmitted immediately. If there was no connection, the data were temporarily stored on the device and automatically sent once connectivity was restored. This mechanism ensured that no monitoring data were lost, even during periods of internet unavailability.

Monitoring data were stored as events with timestamps. When a user applied an exercise, the event included the date and time of the exercise start, the name of the exercise, and the number of repetitions completed. For example, "2023-07-0413:35:34,exercise,"{"name":"Heel raise sitting","number_reps":10}"

Events were also created following this scheme for other activities, such as starting the app, playing a health-literacy game, checking the training progress, or answering the weekly question on falls.

As there is no event in the KOKU monitoring data that indicates the actual end of KOKU usage, the start time of the last event has to be used to calculate the usage time.

From the collected monitoring data events, various usage parameters were calculated, including training days (ie, days with at least 1 recorded exercise event), the number of exercises and repetitions performed, and the total time spent using KOKU (including games, progress tracking, fall questionnaires, etc). All data for each participant were calculated over the entire KOKU usage period and on a weekly basis.

Safety was evaluated by a weekly fall question within KOKU and by documenting clinical events.

Since the FES-I is asked after reaching levels 3 and 6, only the data from participants who have also reached the corresponding level are available for these points in time.

#### Instrumented Physical Capacity and Gait Measurements

The assessments were conducted at the beginning of the KOKU introduction home visit (premeasurement) and after 3 months (postmeasurement).

##### Instrumented Timed Up and Go Test

The sensor-based mTUG system (mTUG, medical device, mHT—mHealth Technologies) provides an instrumented version of the Timed Up and Go Test (TUG), enhancing traditional assessments by providing detailed mobility metrics. This system has been validated in older adults and is certified as a medical device [28]. The mTUG system comprises an inertial sensor (mHT Sensor; mHealth Technologies) featuring a triaxial accelerometer (±2 g range) and a triaxial gyroscope (±250°/s range), with a sampling rate of 100 Hz. This sensor connects to an assessor’s smartphone (Samsung Galaxy S10e; Samsung Group Seoul, Android 5.0.1) via Bluetooth, which is equipped with a customized app that allows the assessor to manually initiate and terminate measurements.

During the assessment, the conventional TUG setup was arranged, including a chair, a 3-m walk, and a turning marker [29]. The assessor attached the inertial sensor to the participant’s lower back using a belt and initiated the test via the mTUG app. Participants performed the TUG test twice at a normal walking pace, with the procedure thoroughly explained beforehand. The first repetition was considered a trial run [18], while only the second repetition was taken into account for the results.

The data collected during the tests were stored on the assessor’s smartphone. Upon completion, the raw sensor data underwent postprocessing using the validated mTUG algorithm [28]. From this processed data, the total duration of the second repetition was extracted. Additionally, the system analyzed and provided durations for specific TUG segments (sit-to-stand, walk 1, turn 1, walk 2, turn 2, stand-to-sit) [30] as well as the number of steps needed for the measurement.

##### Instrumented 30-Second Chair Rise Test

An instrumented version of the original 30-Second Chair Rise Test was used [9]. Participants were seated on a chair with their feet flat on the floor, arms crossed over the chest, and back straight. They were instructed to stand up fully and sit down as many times as possible within 30 seconds while maintaining proper form. A linear encoder (MuscleLab Power model MLPRO; Ergotest Technology) with a sampling frequency of 100 Hz and a resolution of <0.075 mm was used to measure the displacement of a string unrolling from a box over time. The encoder was attached to the participant’s waistband or using a waist belt at the level of the lumbar vertebra L5. In the conventional 30-second CRT, if a participant was in the middle of a rise when time expired, only fully completed repetitions were counted [31]. As a result of the instrumented test execution, in addition to the fully completed repetitions, the proportion of partially executed repetitions in the fully used 30 seconds was analyzed. In addition to the number of repetitions performed, the frequency, and the inactive time during sitting and standing were calculated by the application software (MuscleLab 4010, V8.09; Ergotest Technology).

##### Instrumented Assessment of Real-World Walking

Real-world walking was objectively measured over 7 days using the body-fixed wearable sensor Axivity AX6 (Axivity Ltd). The device was fixed to the lower back (L5) using waterproof adhesive tape. The AX6 is a 6-degrees of freedom inertial measurement unit with the following configuration: a triaxial accelerometer with a range of ±8 g and a resolution of 1 mg, triaxial gyroscope with a range of ±2000 degrees per second and a resolution of 70 milli-degrees per second (milli-degrees per second), and sampling frequency of 100 Hz. A measurement was defined as valid if a wear time of more than 20 hours was detected on at least 3 days [32].

In the first step, walking bouts (WBs) were identified and categorized by their duration (10‐30 s, >30 s). A WB was defined as a walking sequence containing at least 2 consecutive strides of both feet (eg, R–L–R–L–R–L with R/L being the right/left foot contact with the ground) [33].

Based on that, different digital mobility outcomes were extracted within these WBs for further analysis [34,35]. The digital mobility outcome measurements were carried out before randomization into the randomized controlled trial about 4 weeks before starting with KOKU and at postmeasurement.

### Statistical Analysis

Pragmatic considerations, including logistical and financial constraints, limited data collection to a subset of participants from the SMART-AGE study, resulting in a final subsample requirement of 116 participants.

Descriptive statistics, including means and SDs or median and range, were calculated for all outcome measures. The distribution of the variables, including assessments of normality, is presented in Multimedia Appendix 1.

To assess differences between premeasurement and postmeasurement, 2-sided paired *t* tests were conducted. Effect sizes were determined using Cohen *d*, with values interpreted as small (0.2), medium (0.5), and large (0.8) effects [36]. A significance level of *α* less than or equal to .05 was set for all statistical analyses; *P* values were Bonferroni-Holm adjusted for each instrumented measurement, that is, the instrumented Timed Up and Go Test (iTUG), the instrumented 30-Second Chair Rise Test (iCRT), and real-world walking outcomes.

In addition, regression analyses were conducted to identify potential determinants of physical capacity in instrumented assessments. For each instrumented measurement, we chose the variable with the highest effect size to conduct a regression analysis. A change score was calculated by subtracting the premeasurement from the postmeasurement for the total time in the iTUG and the percentage of inactive time in the iCRT, respectively. A negative change score meant a decrease in time, which signifies an improvement in iTUG performance, and a positive change score in iCRT score indicated an improvement. The change score was then entered as the dependent variable in a multiple linear regression model. Since we expected KOKU usage to contribute to an improvement in performance, we included the highest reached KOKU level at postmeasurement and the average days the participant had been active on the app per week as predictors. In addition, we wanted to investigate the influence of baseline performance as well as its interactions with the KOKU usage.

All statistical analyses were performed using the statistical software SPSS (version 29.0; IBM Corp.) and RStudio (version 2023.09.1; Posit Software).

## Results

### Participant Data

In total, data of 113 participants were included in the following analyses (Table 1). The number of dropouts was lower than assumed (n=5, 4.2%). Two participants went on holiday without taking the tablet with them, and 3 participants were excluded because of missing postmeasurement data after 3 months.

**Table 1.** Characteristics of the study sample at baseline^a^^,^^b^^,^^c^.

Characteristics	Values	Available sample
Age (y), mean (SD; range)	74.02 (5.36; 66‐87)	113
Female sex, n (%)	63 (56)	113
Body mass index (kg/m^2^), mean (SD)	26.39 (4.60)	110
German citizenship, n (%)	99 (96)	103
Educational level (y), mean (SD; range)	12.23 (1.98; 7‐16)	109
Living alone, n (%)	41 (39)	104
Retired, n (%)	107 (99)	108
Groll Functional Comorbidity Index (score), mean (SD)	2.18 (1.86)	109
Short Falls Efficacy Scale-International^b^ (score), mean (SD; range)	8.42 (2.32; 7‐18)	113
Fall history (last 12 months)^b^	44 (39)	113
Trail Making Test, mean (SD)		113
Test duration A (s)	47.81 (21.22)	
Test duration B (s)	11.76 (43.18)	
Ratio (B/A)	2.19 (0.73)	
Six-item Cognitive Impairment Test^c^ (score), mean (SD; range)	0.91 (1.37; 0‐4)	113
WHO Quality of Life Scale (score), mean (SD)		
Physical domain	79.41 (15.09)	107
Psychological domain	74.73 (12.47)	107
Social relationships domain	68.22 (14.51)	107
Environment domain	81.36 (11.12)	109
Mobile Device Proficiency Questionnaire (score), mean (SD)	26.63 (9.65)	111

a Data from the SMART-AGE initial assessment, which was applied 1 month before the data collection for this study.

b This was part of the Keep On Keep Up initialization.

c Participants with>7 error points were excluded from the SMART-AGE study due to cognitive impairment.

### KOKU Usage

The average duration between premeasurement and postmeasurement was 95.62 (SD 8.49; range 77‐146 days). Throughout this period, no falls or other adverse events were reported while or due to using KOKU.

### Usage and Activity

Participants trained with KOKU for an average of 24 (SD 15; 1-71 days). On these training days, participants spent an average of 12.90 (SD 7.36; range 0.08‐64.32 min) using KOKU. The average break duration between training days was 3.75 (SD 6.99; range 1‐91 days).

The total number of exercises performed during the study period ranged from 2 to 213 exercises, with a mean of 75.9 (SD 47.3) exercises. On each training day, participants performed an average of 3.17 (SD 0.98; range 1‐18) exercises with 22.1 (SD 24.9; range 0‐250) repetitions for each exercise leading to an average time spent on exercises within KOKU of 6.95 (SD 4.16; range 0.13‐33.03 minutes).

About half of the participants (n=56, 49.6%) had reached level 5 or higher, with the largest proportion at 28.3% (n=32) of the participants reaching the maximum level 7. Sixteen (14.2%) participants did not complete the first level. Out of these, 7 (6.7%) participants did not use KOKU at all.

Regarding the health-literacy games, 67 (59.3%) participants played at least 1 health literacy game within KOKU, playing an average of 7.82 (SD 8.95; range 1‐53) games per participant.

### Weekly Engagement Trends

**Figure 1.**
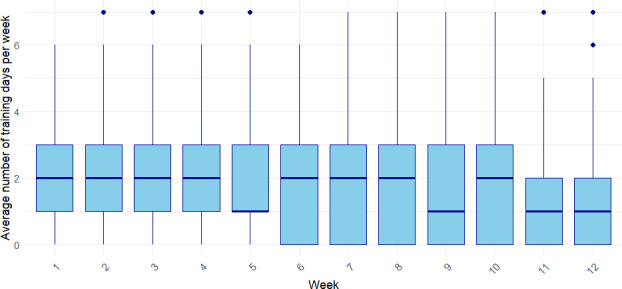
Average number of training days per week over the study period.

**Figure 2.**
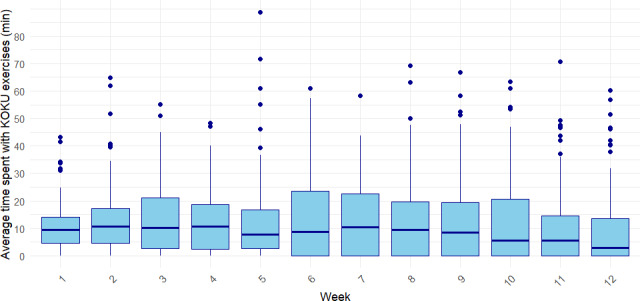
Time spent with Keep On Keep Up (KOKU) exercises per week.

In the first 3 weeks, participants exercised on average more than 2 days per week, with the highest median training frequency observed in weeks 2 and 3 (Figure 1). From week 4 onward, a gradual decline appeared with around 1.5 days per week between weeks 5 and 10, reaching the lowest point in week 12. The total time spent using the KOKU program per week averaged 32.18 (SD 3.48) minutes and showed a slight increase during the initial weeks, peaking at 40.62 minutes in week 10 before declining to 26.45 minutes by week 14. The actual time spent exercising per week followed a similar trend, reaching a maximum of 13.71 minutes in week 10 before decreasing thereafter (Figure 2). The number of exercise repetitions rose from 24.55 in week 1 to 33.85 in week 4 and then remained relatively stable, ranging between 30 and 34 repetitions per week.

#### Short Falls Efficacy Scale-International

Based on established cut-off scores [37], at premeasurement, 81 (71.7%) participants reported low concern about falling (scores 7‐8), 28 (24.8%) had a moderate concern (scores 9‐13), and 4 (3.5%) exhibited a high concern (scores 14‐28). Fear of falling showed a trend toward a 0.7-point reduction in the FES-I. Mean FES-I scores decreased from 8.42 (SD 2.32, range 7-18; n=113) at premeasurement to 8.09 (SD 1.81, range 7-15; n=76) at Level 3 and further to 7.72 (SD 1.39, range 7-13; n=43) at Level 6.

Among those with high concern at premeasurement, 2 participants reached level 3, showing a reduction in their FES-I scores by 2 and 8 points, respectively. One of them progressed to level 6, with a total score reduction of 4 points compared to premeasurement.

### Instrumented Physical Capacity and Gait Measurements

#### Instrumented Timed Up and Go Test

The iTUG analysis in 104 participants revealed a nonmeaningful increase in the total duration from premeasurement (10.30, SD 2.45 s) to postmeasurement (10.71, SD 2.71 s), without a significant difference after alpha adjustment for multiple testing (*t*_103_= 2.65, *P*=.06, *d*=0.26; Table 2).

**Table 2.** Results of the instrumented Timed Up and Go Test (iTUG) (n=104).

Variables	Premeasurement, mean (SD)	Postmeasurement, mean (SD)	*P* value^a^	Cohen *d* (95% CI)
Total duration (2. repetition) (s)	10.30 (2.45)	10.71 (2.71)	.009	0.26 (0.06 to 0.46)
Segments (s)				
Sit to walk	1.19 (0.33)	1.20 (0.29)	.96	0.01 (−0.19 to 0.20)
Walk 1	2.25 (0.90)	2.46 (0.99)	<.001	0.39 (0.19 to 0.59)
Turn 1	2.34 (0.52)	2.35 (0.46)	.63	0.05 (−0.14 to 0.24)
Walk 2	1.48 (0.63)	1.66 (0.90)	.006	0.28 (0.08 to 0.47)
Turn 2	2.03 (0.41)	2.03 (0.33)	.94	0.01 (−0.18 to 0.20)
Turn to sit	3.03 (0.78)	3.05 (0.63)	.80	0.03 (−0.17 to 0.22)
Further parameters				
Total number of steps	10.41 (1.92)	10.79 (2.18)	.02	0.23 (0.04 to 0.43)

a *P* values are Bonferroni-Holm adjusted and for *t *tests.

Regarding the TUG segments, walk 1 duration increased significantly between premeasurement (2.25, SD 0.90) and postmeasurement (2.46, SD 0.99) measurement (*t*_103_=3.96, *P*<.001, *d*=0.39). No statistically significant differences were observed for the other segments. The total number of steps increased from 10.4 (SD 1.9) to 10.8 (SD 2.2; *t*_103_=2.39, *P*=.02, *d*=0.23).

The multiple regression model accounted for 15% of the variance in change scores (*F*_5,104_=4.86, *P*<.001, *R^2^*=0.15) with all predictors and interaction terms having a significant contribution (supplementary 5 in Multimedia Appendix 1). Negative effects mean a reduction in change score, which can represent a stabilization or improvement. A higher average of weekly training days was associated with a reduction in change score (*β*=−3.18, *t*_104_=−3.66; *P*<.001). The longer the iTUG time at baseline, the smaller the change score (*β*=−.55, *t*_104_=−2.53; *P*=.01), which suggests that participants with worse physical capacity at baseline could benefit more from training with KOKU. The interaction between baseline performance and average weekly training days was positive (*β*=−3.56, *t*_104_=3.82; *P*<.001), implying that participants with lower baseline performance who trained more frequently showed increased change scores, thereby reflecting less improvement.

Although the highest KOKU level achieved was independently associated with increased change scores (*β*=2.85, *t*_*104*_=4.07; *P*<.001), its interaction with baseline performance was negative and significant (*β*=−3.25, *t*_104_=−4.39; *P*<.001).

#### Instrumented 30-Second Chair Rise Test

A total of 91 participants had valid premeasurement and postmeasurement data for the iCRT (Table 3).

**Table 3.** Results of the instrumented 30-Second Chair Rise Test (iCRT) (n=91).

Variables	Premeasurement, mean (SD)	Postmeasurement, mean (SD)	*P* value^a^	Cohen *d* (95% CI)
Number of fully completed repetitions	10.98 (2.41)	11.63 (2.92)	.04	0.21 (0.01 to 0.42)
Number of repetitions based on the complete 30 seconds	11.37 (2.41)	12.05 (2.97)	.03	0.23 (0.02 to 0.43)
Frequency (repetitions/second)	0.38 (0.08)	0.40 (0.10)	.03	0.23 (0.01 to 0.43)
Percentage of inactive time^b^	13.86 (8.27)	8.46 (5.07)	<.001	−0.60 (−0.82 to −0.37)

a *P* values are Bonferroni-Holm adjusted and for *t* tests.

b Inactive time between sit-to-stand and stand-to-sit, respectively.

The number of fully completed repetitions (*t*_90_=2.04, *P*=.04, *d*=0.21), the number of repetitions based on the full 30-second duration (*t*_90_=2.16, *P*=.03, *d*=0.23) as well as the frequency of repetitions (*t*_90_=2.156, *P*=.03, *d*=0.23) increased. A significant reduction was observed in the percentage of inactive time (*t*_90_=−5.70, *P*<.001, *d*=−0.60).

The multiple regression model for the iCRT explained 70% of the variance (*F*_6,84_=36.45, *P*<.001, *R*^2^=0.7; supplementary 6 in Multimedia Appendix 1). Of all included predictors, only baseline performance showed a significant association with the change in the percentage of inactive time (*β*=−1.12, *t*_84_=−8.0; *P*<.001). Participants with a higher percentage of inactive time at baseline had smaller change scores, indicating that they showed more improvement. This factor alone could account for 69% of the variance in change scores. Our chosen measures of KOKU usage did not influence improvements in the iCRT.

#### Instrumented Assessment of Real-World Walking

The analysis of real-world walking measures in 91 participants showed no significant changes in most parameters between premeasurement and postmeasurement (Table 4). Notably, significant reductions were observed in walking speed in longer bouts (>30 s), with the average speed decreasing from 0.85 to 0.83 m/s (*d*=0.46; *P*<.001) and the 90th percentile walking speed declining from 1.07 to 1.04 m/s (*d*=0.49; *P*<.001), while overall walking patterns remained unchanged.

**Table 4.** Results of the real-world walking measures (n=91).

Variables	Premeasurement, mean (SD)	Postmeasurement, mean (SD)	*P* value^a^	Cohen *d* (95% CI)
Daily walking duration (min)	103.45 (38.82)	103.49 (40.47)	.98	−0.003 (−0.21 to 0.21)
Total number of WBs^b^	350.92 (123.97)	349.54 (119.19)	.71	0.04 (−0.17 to 0.25)
Number of WBs <10 s	160.21 (62.40)	159.92 (60.13)	.89	0.01 (−0.19 to 0.22)
Number of WBs 10‐30 s	128.47 (48.63)	127.33 (46.25)	.48	0.07 (−0.13 to 0.28)
Number of WBs >30 s	31.74 (17.30)	32.58 (17.02)	.19	−0.14 (−0.35 to 0.07)
Average walking speed in WBs >30 s (m/s)	0.85 (0.13)	0.83 (0.13)	<.001	0.46 (0.24 to 0.68)
90th percentile of walking speed in WBs >30 s (m/s)	1.07 (0.19)	1.04 (0.18)	<.001	0.49 (0.27 to 0.71)

a *P* values are Bonferroni-Holm adjusted and for *t* tests.

b WB: walking bout.

About 15% of the variance in the change of 90th percentile speed for the 30-second WBs could be accounted for in the multiple regression model (*F*_5,85_=2.94, *P*=.01, *R*^2^= 0.15; supplementary 7 in Multimedia Appendix 1). There were no significant main or interaction effects of the chosen predictors on the change in average speed.

## Discussion

### Principal Findings

The aim of this study was to evaluate the extent to which KOKU was implemented as intended. This process evaluation assessed both adherence to the protocol and participant engagement. As a digital intervention, KOKU represents a complex health care initiative that requires process evaluation to determine the fidelity and quality of its implementation, as well as the influence of contextual factors. It was anticipated that KOKU might require adaptation across different settings or populations and that the evaluation would reveal both strengths and weaknesses in its delivery.

While pre-post outcome measures were included to explore potential changes over time, this study was not designed to provide definitive evidence of effectiveness. Therefore, the observed outcome trends should be interpreted as exploratory and descriptive rather than causal. The combination of implementation-focused evaluation with preliminary outcome data provides insights into how the intervention was delivered and received, while also generating hypotheses for future controlled effectiveness studies.

No falls or other adverse events were reported by the KOKU participants, indicating high program safety. These aspects are of indispensable importance for any digital intervention targeting populations in unsupervised home settings.

Participants engaged with KOKU on 2 to 3 training days per week, which is in line with the recommendations [14]. Engagement levels varied among participants, with some using the system only once while others used KOKU extensively. General adherence trends suggest that engagement with KOKU showed good initial engagement in the first 3 weeks, followed by a gradual decline over time. This pattern of declining engagement is consistent with previous research on digital health interventions [38]. However, despite this decline, the total number of exercises performed remained relatively stable, which could suggest that those who continued using KOKU maintained a consistent level of exercise activity. Moreover, the increase in exercise repetitions in the first 4 weeks, followed by stability, reflects the observed usage pattern in the logged training data rather than indicating a specific behavioral mechanism. As no subjective measures (eg, participant-reported reasons for engagement or disengagement) were collected, the underlying reasons for these trends cannot be determined. However, the collection of such subjective data is currently being implemented in a follow-up study with KOKU to better understand factors influencing user engagement and adherence [39].

The time spent on KOKU exercises remained relatively short. The recommendation by the KOKU developers is at least 10 minutes per session for inactive older adults with prefrailty to moderate frailty. In comparison, the conventional Otago program recommends training sessions of 30 minutes or more [30]. This limited training duration may have contributed to the limited improvements, alongside recruiting more active older adults.

By the end of the study, nearly half of the participants had reached level 5. To reach this level, participants had to use KOKU regularly for at least 8 weeks without feeling unstable, indicating that a substantial proportion of users demonstrated sustained engagement and progression in their training.

A substantial proportion of participants did not complete the first level or did not use KOKU at all, highlighting potential barriers to engagement. It is important to note that the study cohort was relatively fit at baseline, as indicated by the motor assessment data reported [40]. This characteristic is likely related to the study’s recruitment criteria, which required a certain level of digital literacy to participate [19]. This requirement may have introduced a selection bias toward individuals who were healthier, more functionally independent, and potentially more motivated than the general target population. As a result, this may have led some participants to perceive KOKU as insufficiently challenging, resulting in minimal or no use. However, other barriers such as individual differences in motivation, usability challenges, or others may also have played a role.

### Instrumented Measurements

Compared to traditional analog assessments, which often rely on subjective interpretation, instrumented measurements offer a more comprehensive and standardized way of capturing movement data. This can be particularly valuable in clinical and research contexts where even small changes might indicate early functional decline or emerging effects of an intervention such as KOKU.

The iTUG results indicated an increase in total time needed to perform 1 repetition. This increase in duration may reflect performance variability rather than a meaningful decline in capacity. Analysis of individual gait segments showed no significant differences between premeasurement and postmeasurement. Further investigation is warranted to determine whether the observed trend of an increase in the number of steps during the iTUG represents a robust effect that reflects a compensatory adaptation or a trial-to-trial variability. Overall, the iTUG results suggest that mobility performance measured with the iTUG remained relatively stable between premeasurement and postmeasurement, with only minor variations that are unlikely to be of significant clinical concern. Future studies could explore whether these small changes persist over time or whether they are influenced by factors such as fatigue, learning effects, or external conditions.

The results of the iCRT indicate that participants performed slightly better in postmeasurement compared to premeasurement, as evidenced by small increases in the number of fully completed repetitions, the number of repetitions based on the complete 30 seconds, and the frequency of repetitions. However, these differences were small and are unlikely to represent clinically meaningful improvements. Rather, they may reflect minor variations in performance, familiarization with the task, or day-to-day variability. The most notable finding was the reduction in inactive time, with a medium effect size. This suggests that, during the postmeasurement, participants spent less time pausing or resting between sit-to-stand and stand-to-sit transitions. While this change may indicate a difference in movement execution, its clinical relevance remains uncertain in this sample. The iCRT findings highlight the importance of considering not just repetition counts as in conventional 30-second CRT, but also more detailed metrics of movement execution, such as inactivity between movement segments. Future research should explore whether the observed reduction in inactive time during iCRT translates into clinically relevant functional improvements in different populations, particularly in rehabilitation settings or frail populations. Additionally, investigating potential factors influencing inactivity, such as fatigue, motivation, or motor control, could provide further insights into optimizing performance.

In line with the capacity measures, the real-world walking measures revealed minimal changes between premeasurement and postmeasurement across most parameters, indicating that participants’ everyday mobility patterns remained largely stable. A small reduction in walking speed during walking bouts of >30 seconds was observed; however, this change was small and unlikely to reflect a clinically meaningful decline in functional mobility [41].

Overall, changes in both capacity and real-world measures were minimal, likely due to the characteristics of our study sample. About 82% (n=93) of the participants performed below the 12-second threshold in the TUG, reflecting overall good mobility [42]. Their real-world walking metrics also aligned with those of healthy community-dwelling older adults from the inCHIANTI cohort [40]. This relatively high baseline fitness may also help to contextualize the small observed changes in motor outcomes. Larger effects might be expected in a frailer population with more potential for improvement through KOKU usage.

Nevertheless, the use of instrumented assessments to analyze premeasurement to postmeasurement changes revealed more nuanced and detailed insights that cannot be captured by traditional analog methods. The findings open up new avenues for understanding changes in motor strategies and highlight the potential of instrumented analysis to uncover early or otherwise hidden shifts in performance.

The regression analyses examined associations between KOKU usage, baseline performance, and changes in functional mobility outcomes. In the absence of a control group, causal interpretations are not possible, and the findings should be interpreted as exploratory associations.

For the iTUG, the multiple regression model explained a modest but significant proportion of the variance in change scores. Higher KOKU training frequency was associated with greater improvement in iTUG performance. Additionally, participants with poorer baseline performance tended to show larger improvements, which may reflect greater potential for change in this group.

In contrast, the iCRT model showed stronger predictive capacity for changes in inactive time, a key indicator of movement efficiency. Here, only baseline performance predicted change: participants with more inactive time at baseline tended to show greater improvements. KOKU usage variables (ie, training frequency and level reached), however, showed no significant associations with iCRT outcomes.

### Future Perspectives

In the future, the variety of KOKU exercises should be expanded, including stratified progression and adaptive difficulty, allowing users with different levels of balance and strength to train at an appropriate level and achieve further progress.

In this study, all instrumented measurements were carried out by assessors. In the real world, however, digital interventions that can be used unsupervised and independently need to be combined with digital self-assessments to provide a comprehensive digital prevention strategy. An iTUG self-assessment could serve as an orientation tool to guide exercise selection rather than as a strict eligibility criterion. For example, users with slower iTUG performance (>12  s) could be guided to exercises targeting measurable improvement, while fitter users (<12  s) might benefit primarily from maintaining mobility and functional capacity. Adaptive difficulty would enable these users to remain challenged without expecting large gains in performance, supporting long-term function and engagement.

The iTUG self-assessment could be repeated on a regular basis, for example, every 3 months, to enable self-monitoring and evaluation of training progress, as well as to adjust exercise selection accordingly. The mTUG medical device system used in this study, however, is designed for supervised measurements in clinical or laboratory settings and not recommended for self-assessment. A recent study demonstrated that a smartphone-based iTUG self-test (Up&Go app) yields comparable results to the validated mTUG system with excellent concurrent validity and test-retest reliability [18]. Five test rounds are performed within this app, allowing users to familiarize themselves with the test procedure and minimizing potential variability in test performance due to initial uncertainty in self-assessment [18]. The Up&Go app is freely available for download on all Android and iOS smartphones in German-speaking countries. It was co-designed with older adults and includes comprehensive information and video instructions for independent test preparation and execution [43]. From our point of view, the integration of a self-assessment into KOKU usage would add considerable value to increase the target group-intervention fit and improve users’ self-management. Similar types of self-assessment would also be conceivable for the iCRT.

### Limitations

Several limitations should be acknowledged. The effect sizes for most performance metrics were small; they did not reach the level of minimal important differences. By definition, this process evaluation is not a causal interpretation regarding the effects of the KOKU training. The study did not investigate the underlying reasons for declining engagement, which could be influenced by personal motivation, external life factors, or the perceived difficulty of the exercises. In addition, activities outside of KOKU—such as training in a sports club—were not recorded. Gaining qualitative insights into user experiences and exercise habits beyond KOKU usage could offer a deeper understanding of the factors that facilitate or hinder engagement. As part of a single-blinded randomized controlled trial, this was not feasible in this study. While instrumented assessments offer numerous advantages, they are also prone to technical errors. Incorrectly recorded data may only be recognized retrospectively and cannot always be reconstructed. This issue occurred with the iCRT and the real-world measurements, resulting in a reduced sample size.

### Conclusions

This study demonstrated that KOKU is a feasible and safe digital training intervention for older adults, offering an accessible way to empower them to independently engage in balance and strength exercises at home. While initial engagement was strong, usage declined after 3 weeks. This might require a phone call or teleconsultation to improve mid-term adherence.

The study sample consisted mainly of individuals with good physical capacity and function. With this in mind, the small changes in physical capacity and real-world walking highlight the importance of better tailoring of such interventions to individual levels of physical capacity, with potential improvements through more challenging exercises. These findings may support a stratified training approach with adaptive difficulty levels. Instrumented assessments provided valuable insights. Moreover, combining digital interventions with self-assessments, such as iTUG self-testing, could enhance user autonomy and optimize the fit of the intervention to individual capacities. Overall, digital training interventions, such as KOKU, have the potential to complement traditional care models, offering older adults an effective means to maintain their mobility, independence, and quality of life.

## Supplementary material

10.2196/80372 Multimedia Appendix 1 Distribution of the Keep On Keep Up (KOKU) usage data and of the variables of the instrumented Timed Up and Go Test, the instrumented Chair Rise Test, and the sensor-based real-world walking.

## Abbreviations

CONPHES: Consensus-Based Process Evaluation Reporting Guideline for Public Health Intervention Studies
FES-I: Falls Efficacy Scale-International
iCRT: instrumented 30-Second Chair Rise Test
iTUG: instrumented Timed Up and Go Test
KOKU: Keep On Keep Up
TUG: Timed Up and Go Test
WB: walking bout
