# Supplementary material for: Usage of the Tablet-Based “Keep On Keep Up” Digital Program and Resulting Changes in Physical Capacity and Real-World Walking in Community-Dwelling Older Adults: Process Evaluation
Source: JMIR Form Res. 2026 May 13;10:e80372. doi: 10.2196/80372 (PMC13170663; doi:10.2196/80372)
Supplement: Multimedia Appendix 1 [file formative-v10-e80372-s001.docx]

Supplementary 1: KOKU usage data

**Total number of training days**

**Total number of exercises**

**Total number of games**


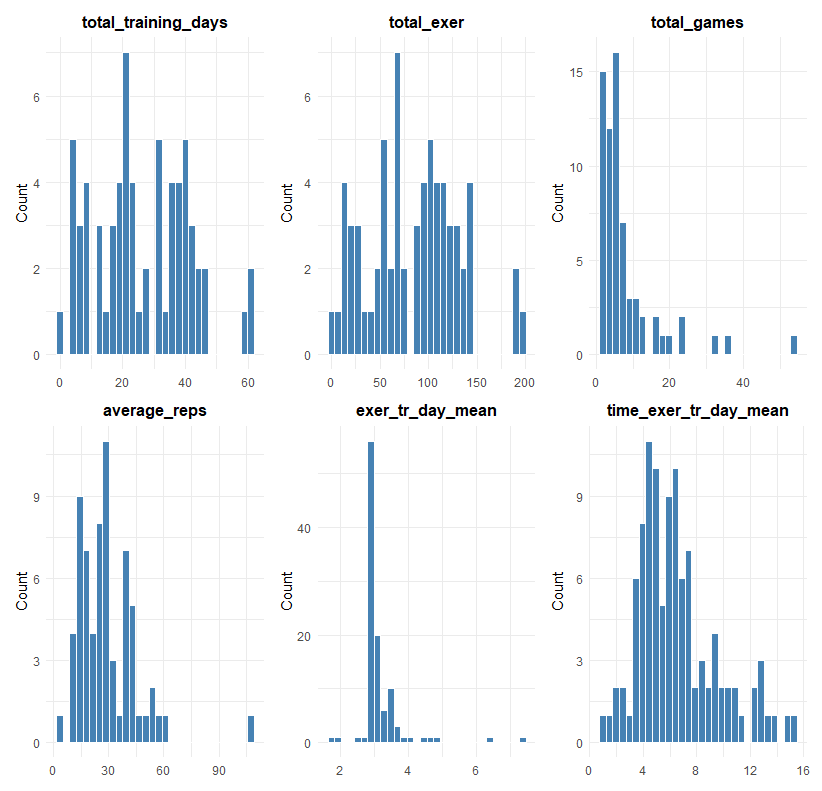


**Average time spent with exercises**

**Average exercises per training day**

**Average repetitions per exercises**

Supplementary 2: Distribution of the variables of the instrumented Timed Up and Go Test


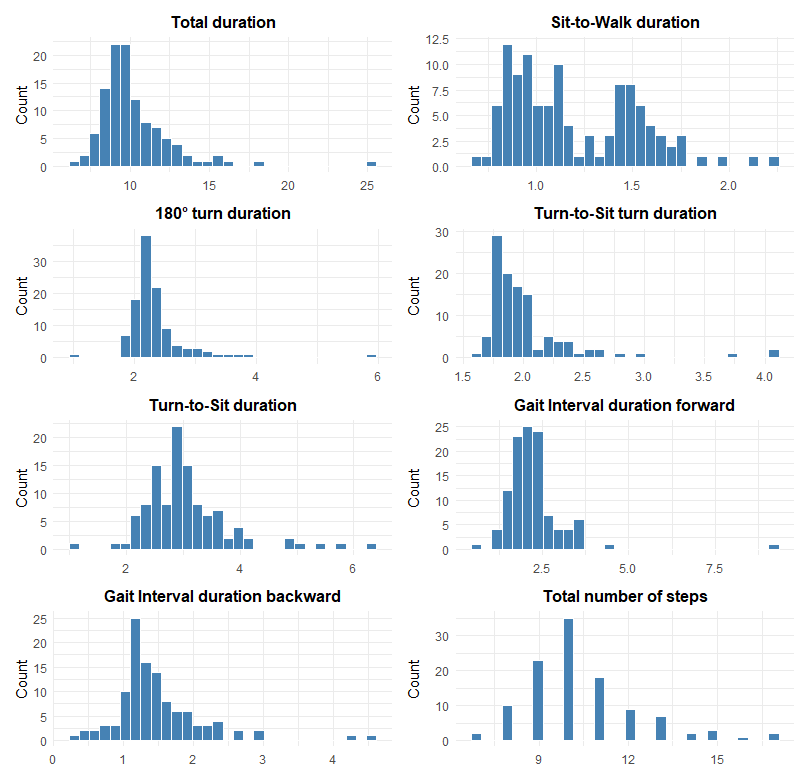


**Turn to Sit**

**Turn 2**

**Turn 1**

**Sit to walk**

**Total duration**

**Walk 2**

**Walk 1**

**Total number of steps**

Supplementary 3: Distribution of the variables of the instrumented Chair Rise Test

**Number of fully completed repetitions**

**Number of repetitions based on the complete 30 sec**


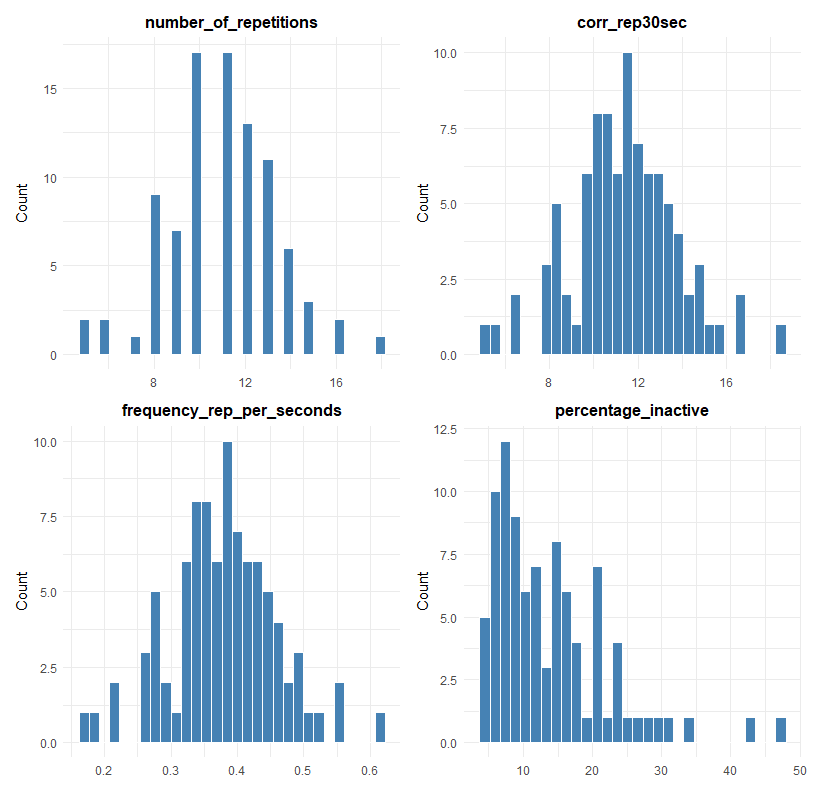


**Percentage of inactive time**

**Frequency (repetitions(sec)**

Supplementary 4: Distribution of the sensor-based real-world walking variables

**Daily walking duration**


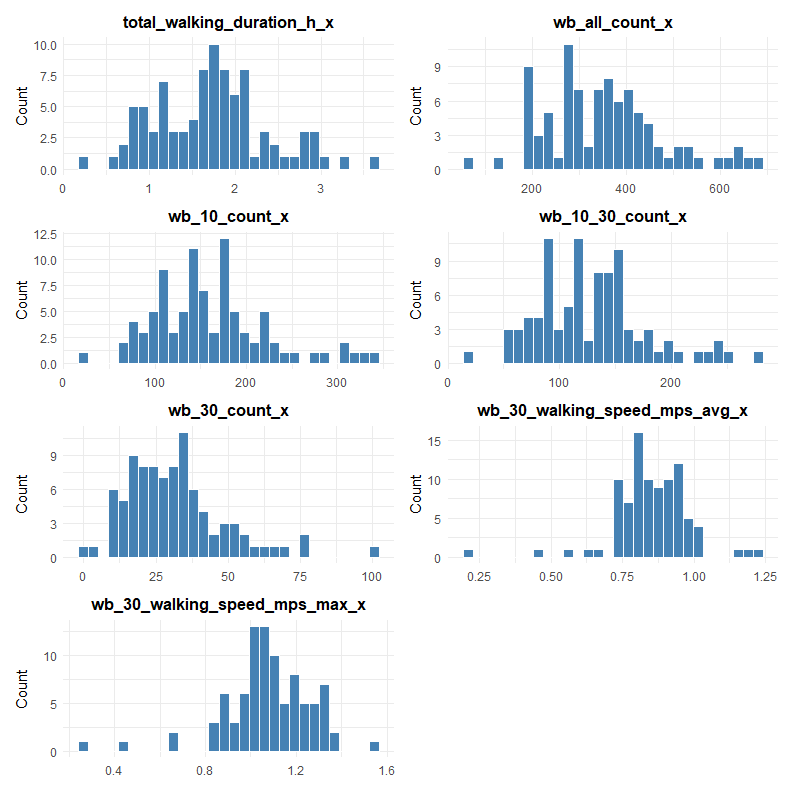


**90th percentile of walking speed in WBs > 30s**

**Average walking speed in WBs >30s**

**Number of walking bouts >30sec**

**Number of walking bouts 10-30sec**

**Number of walking bouts <10sec**

**Total number of walking bouts**

Supplementary 5: Regression of change score of the instrumented Timed Up and Go test

|  |  |  | 95% Conf. Int | |  |  |  |
| --- | --- | --- | --- | --- | --- | --- | --- |
|  | Estimate | SE | 2.5% | 97.5% | β | t-value | p-value |
| (Intercept) | 3.991 | 1.487 | 1.042 | 6.940 |  | 2.684 | 0.008 |
| Mean active days | -5.304 | 1.448 | -8.176 | -2.433 | -3.179 | -3.663 | <0.001 |
| KOKU level | 2.063 | 0.507 | 1.059 | 3.068 | 2.847 | 4.073 | <0.001 |
| Total duration HB3 | -0.356 | 0.141 | -0.635 | -0.077 | -0.553 | -2.533 | 0.01 |
| KOKU level x Total duration HB3 | -0.205 | 0.05 | -0.298 | -0.113 | -3.249 | -4.389 | <0.001 |
| Mean active days x Total duration HB3 | 0.530 | 0.139 | 0.255 | 0.805 | 3.556 | 3.820 | <0.001 |
| Note. R^2^_adj_ = 0.151 (N= 110, F(5, 104) = 4.861, p < 0.001) | | | | | | | |

Supplementary 6: Regression of change score of the instrumented chair rise test (inactive time)

|  |  |  | 95% Conf. Int | |  |  |  |
| --- | --- | --- | --- | --- | --- | --- | --- |
|  | Estimate | SE | 2.5% | 97.5% | β | t-value | p-value |
| (Intercept) | 11.426 | 2.490 | 6.476 | 16.376 |  | 4.589 | <0.001 |
| Mean active days | -0.703 | 1.886 | -4.454 | 3.047 | -0.074 | -0.373 | 0.710 |
| KOKU level | -0.796 | 0.793 | -2.374 | 0.781 | -0.194 | -1.004 | 0.318 |
| Total duration HB3 | -1.245 | 0.151 | -1.546 | -0.945 | -1.142 | -8.234 | <0.001 |
| KOKU level x Total duration HB3 | 0.006 | 0.052 | -0.098 | 0.110 | 0.025 | 0.116 | 0.908 |
| Mean active days x Total duration HB3 | 0.180 | 0.136 | -0.089 | 0.450 | 0.352 | 1.329 | 0.187 |
| Note. R^2^_adj_ = 0.702 (F(5,85) = 43.42, N= 91, p < 0.001) | | | | | | | |

Supplementary 7: Regression of change score of real-life walking (90^th^ percentile of walking speed in WBs > 30s)

|  |  |  | 95% Conf. Int | |  |  |  |
| --- | --- | --- | --- | --- | --- | --- | --- |
|  | Estimate | SE | 2.5% | 97.5% | β | t-value | p-value |
| (Intercept) | -0.014 | 0.086 | -0.184 | 0.156 |  | -0.162 | 0.871 |
| Mean active days | -0.010 | 0.076 | -0.162 | 0.141 | -0.161 | -0.137 | 0.891 |
| KOKU level | 0.026 | 0.034 | -0.041 | 0.094 | 0.876 | 0.768 | 0.444 |
| Total duration HB3 | -0.036 | 0.079 | -0.192 | 0.120 | -0.107 | -0.461 | 0.646 |
| KOKU level x Total duration HB3 | -0.012 | 0.031 | -0.074 | 0.049 | -0.509 | -0.412 | 0.682 |
| Mean active days x Total duration HB3 | -0.004 | 0.069 | -0.142 | 0.133 | -0.078 | -0.061 | 0.952 |
| Note. R^2^_adj_ = 0.097 (F(5,85) = 2.936, N= 91, p = 0.017) | | | | | | | |
